# External Validation of the Garvan Nomograms for Predicting Absolute Fracture Risk: The Tromsø Study

**DOI:** 10.1371/journal.pone.0107695

**Published:** 2014-09-25

**Authors:** Luai A. Ahmed, Nguyen D. Nguyen, Åshild Bjørnerem, Ragnar M. Joakimsen, Lone Jørgensen, Jan Størmer, Dana Bliuc, Jacqueline R. Center, John A. Eisman, Tuan V. Nguyen, Nina Emaus

**Affiliations:** 1 Department of Health and Care Sciences, Faculty of Health Sciences, UiT – The Arctic University of Norway, Tromsø, Norway; 2 Osteoporosis & Bone Biology Program, Garvan Institute of Medical Research, Sydney, Australia; 3 Department of Clinical Medicine, Faculty of Health Sciences, UiT – The Arctic University of Norway, Tromsø, Norway; 4 Medical Clinic, University Hospital of Northern Norway, Tromsø, Norway; 5 Department of Radiology, University Hospital of Northern Norway, Tromsø, Norway; 6 Department of Endocrinology, St Vincent’s Hospital, Sydney, Australia; 7 School of Medicine Sydney, University of Notre Dame Australia, Sydney, Australia; 8 St. Vincent’s Clinical School, UNSW Australia, Sydney, Australia; 9 School of Public Health and Community Medicine, University of New South Wales, Sydney, Australia; 10 Centre for Health Technologies, University of Technology, Sydney, Australia; Harvard Medical School, United States of America

## Abstract

**Background:**

Absolute risk estimation is a preferred approach for assessing fracture risk and treatment decision making. This study aimed to evaluate and validate the predictive performance of the Garvan Fracture Risk Calculator in a Norwegian cohort.

**Methods:**

The analysis included 1637 women and 1355 aged 60+ years from the Tromsø study. All incident fragility fractures between 2001 and 2009 were registered. The predicted probabilities of non-vertebral osteoporotic and hip fractures were determined using models with and without BMD. The discrimination and calibration of the models were assessed. Reclassification analysis was used to compare the models performance.

**Results:**

The incidence of osteoporotic and hip fracture was 31.5 and 8.6 per 1000 population in women, respectively; in men the corresponding incidence was 12.2 and 5.1. The predicted 5-year and 10-year probability of fractures was consistently higher in the fracture group than the non-fracture group for all models. The 10-year predicted probabilities of hip fracture in those with fracture was 2.8 (women) to 3.1 times (men) higher than those without fracture. There was a close agreement between predicted and observed risk in both sexes and up to the fifth quintile. Among those in the highest quintile of risk, the models over-estimated the risk of fracture. Models with BMD performed better than models with body weight in correct classification of risk in individuals with and without fracture. The overall net decrease in reclassification of the model with weight compared to the model with BMD was 10.6% (*p* = 0.008) in women and 17.2% (*p* = 0.001) in men for osteoporotic fractures, and 13.3% (*p* = 0.07) in women and 17.5% (*p* = 0.09) in men for hip fracture.

**Conclusions:**

The Garvan Fracture Risk Calculator is valid and clinically useful in identifying individuals at high risk of fracture. The models with BMD performed better than those with body weight in fracture risk prediction.

## Introduction

Osteoporotic fractures are an important public health problem. With increasing aging populations, their number will increase placing an additional burden on individuals and society in terms of functional limitations, morbidity, mortality, and costs [1]–[3]. Individuals with high fracture risk are those who can effectively benefit from preventive measures and pharmaceutical interventions and therefore need to be identified in clinical settings. The tools used to identify persons with increased fracture risk have been expanded to rely not only on bone mineral density (BMD) measurements but also to include informative clinical risk factors. Absolute risk or individualized prognosis is considered to be a preferred approach in the assessment of fracture risk and treatment decision making. Several prediction models and tools have been developed to calculate absolute fracture risk. These tools vary according to the number and type of fracture risk factors included, and on the complexity of fracture risk computation [4], [5]. Systematic reviews highlighted that simple tools performed as well as complex tools [5]–[7]. The Garvan Fracture Risk Calculator (www.fractureriskcalculator.com) was stated as one of the simplest tools for fracture prediction developed in a population-based setting applying proper methodology [5]. It is based on data from the Dubbo Osteoporosis Epidemiology Study (DOES) and integrates sex, age, BMD (or body weight), and history of prior fracture and falls into the nomograms. It includes two nomograms; one for prediction of absolute risk for hip fracture and another for any fragility fracture [8], [9]. These nomograms predict the individualized 5-year or 10-year absolute fracture risk for both women and men.

Assessment of the performance of prognostic models in different populations is necessary [4], [10], [11]. The Garvan Fracture Risk Calculator was examined in independent cohorts [11]–[15] and performed well in predicting fracture. However, these validation studies compared the nomograms with other prediction tools, and did not compare the predictive performance between the model with BMD and the model with body weight.

Norway has the highest incidence of hip fractures in the world [16]. Therefore, identification of those at high risk of fracture is warranted, and tools that can be used readily in clinical settings are definitely needed. The present study was designed to evaluate and validate the performance of the Garvan nomograms for predicting 5-year and 10-year risk of fragility fracture in an independent Norwegian cohort of women and men.

## Methods

### Study population

The Tromsø Study [17] is a longitudinal population-based multipurpose study focusing on lifestyle-related diseases. The first survey was conducted in 1974, with repeated surveys in 1979/80, 1986/87, 1994/95, 2001/02 and 2007/08. The fifth survey in 2001/02 (Tromsø 5) invited all persons living in Tromsø between 55–74 years of age and a randomly selected (5–10%) sub-set of women and men in the age groups 25–54 and 75–84 years, who had participated in the second visit of the fourth survey (Tromsø 4) in 1994/95. Of 10,353 persons invited to the first visit of Tromsø 5, 8,130 (79%) attended, and among them, a preselected random sample of 6,969 persons were invited for a second visit one month later, and 5,939 (85%) attended. At the second visit, hip BMD was measured in 3,094 women and 2,132 men, all of whom had one or both hips without nails or prostheses.

Women (n = 2256) and men (n = 1702) aged 60 years or older were selected in order to examine the nomograms performance in a population of similar age as the population in which the nomograms were developed. Of these, 1637 women and 1355 men (aged 60+ years) were included in this analysis. Subjects with missing data were excluded; 603 subjects with missing history of fall and/or previous fracture, 98 subjects with invalid BMD measurements, 8 subjects with pathological fractures, 85 subjects using bisphosphonates, and 184 women using hormone therapy (numbers are overlapping).

The Regional Committee of Medical Research Ethics and the Norwegian Data Inspectorate approved the study. All participants gave written informed consent.

### Questionnaires and measurements

Two self-administered questionnaires were completed by the participants, one before entering the survey, and the other between the two visits of the survey. The questionnaires covered, among others, history of previous fractures, history of falls in the last 12 months, and use of medications. Height and weight were measured to the nearest centimetre/half kilogram whilst wearing light clothing and no shoes.

Dual hip BMD expressed as g/cm^2^ was measured by DXA (GE Lunar Prodigy, LUNAR Corporation, Madison, WI, USA). The scans were performed by specially trained technicians according to the manufacturer provided protocol. The short term in vivo precision error was 1.7% and 1.2% for femoral neck and total hip measurement, respectively, and daily phantom measurements were stable throughout the survey. All scans were reviewed and reanalysed if necessary [18]. Technically incorrect scans and scans of hips with severe deformities were excluded. Scans of the left hip were used for analyses but, if the left hip measurement was ineligible, the right hip scan was used.

### Fracture registration

The fracture registry covered the 15-year period from the date of examination in Tromsø 4 (1994/95) through December 31^st^ 2009 with respect to all non-vertebral fractures. Vertebral fractures were excluded, as date of occurrence for vertebral fractures are not reliable. The fracture registry is based on the radiological archives at the University Hospital of North Norway in Tromsø. The nearest alternative radiology service or fracture treatment facility is located 250 km from Tromsø. The only fractures that would be missed are those, for which no radiology was performed or where such investigations occurred while the subject was travelling and without any subsequent local follow-up examination. The computerized records in the radiological archives of the University Hospital contain the national personal identification number (unique for each resident of Norway), time of investigation, fracture codes and descriptions. All abnormal radiological examinations were reviewed to ascertain the fracture code, to identify exact fracture type and anatomical location, to distinguish consecutive fracture occasions in the same person, and to capture fractures that had not been coded correctly as fractures. In addition, the hospital discharge records were checked with respect to hip fractures. A similar registration has previously been described and validated [19].

### Statistical analysis

Fractures were classified as hip or non-vertebral osteoporotic fractures. The latter included all non-vertebral fractures except fractures of the finger, toe, or skull. Descriptive statistics of the study cohort are presented by sex and fracture status. Comparison of women and men with and without fracture were performed using T-test for continuous variables and chi-square test for categorical variables. Follow-up time was assigned from the date of the BMD measurement at Tromsø 5 (in 2001/02) for each participant, to date of first fracture, migration, death, or to December 31^st^ 2009. Incidence rates (per 1000 person-years) were calculated by dividing the total number of first incident fractures by the sum of person-years during the follow-up period.

The Garvan Fracture Risk Calculator (Appendix S1) estimates the 5-year and 10-year risks of fracture for an individual based on the individual’s risk profile which includes gender, age, bone mineral density (or body weight), frequency of falls during the past 12 months, and the frequency of prior fractures [8], [9]. Two models were used; the first model included BMD, age, prior fracture and fall; the second model replaced BMD with body weight. The prognostic discrimination - between those who suffered a fracture and those who did not - of the models was assessed by the area under the receiver operating characteristics curve (AUC). The predictive accuracy (calibration) of the two models was assessed by the concordance index [20], where the concordance between quintiles of observed and predicted risk of fracture was used as a measure of fit. Moreover, ratios of the predicted fracture risk between those with and without fracture were calculated as back transformation of the log values of the predicted risk difference. Reclassification analysis [21] was used to compare the prognostic performance between the two models. In this analysis, the net reclassification improvement (NRI) for fracture prediction was calculated as the sum of differences in proportions of subjects with fracture and proportions of subjects without fracture who were correctly reclassified with higher/lower risk, between the model with BMD and the model with weight, where positive values would indicate better performance of the model with weight or vice versa. The quartiles of the predicted risk from both models were used as thresholds for the risk groups in the reclassification analysis.

The analyses were performed using the SAS statistical package, v9.2 (SAS Institute Inc., Cary, NC, USA), STATA 12.0 (StataCorp. 2011. Stata Statistical Software: Release 12. College Station, TX: StataCorp LP), and R (R core team 2012). The criterion for statistical significance was set at *p*<0.05.

## Results

Among 1637 women, 356 suffered non-vertebral osteoporotic fractures including 88 hip fractures (mean follow-up 6.9 years). Among 1355 men, 117 suffered non-vertebral osteoporotic fractures where 47 of them were hip fractures (mean follow-up 7.1 years). During the first 5 years of follow-up, 210 women suffered non-vertebral fractures (42 hip) and 68 men suffered non-vertebral fractures (24 hip). The incidences per 1000 person-years of non-vertebral osteoporotic and hip fractures during the follow-up were, respectively, 31.5 (95% Confidence Interval (CI) 28.3–34.9) and 8.6 (95% CI 7.0–10.6) in women, and 12.2 (95% CI 10.2–14.6) and 5.1 (95% CI 3.8–6.7) in men. The baseline characteristics of the study cohort are shown in Table 1.

**Table 1:** Baseline characteristics of women and men. The Tromsø Study.

	Non-fracture	Non-vertebral osteoporotic fractures			
Variables		Any	*p*-value*	Hip	*p*-value*
**Women**	**(n = 1281)**	**(n = 356)**		**(n = 88)**	
Age (y)	69.0 (6.3)	70.3 (6.3)	0.001	74.1 (6.3)	<0.001
Height (cm)	160.3 (6.0)	160.9 (5.9)	0.09	160.6 (6.1)	0.74
Weight (kg)	69.5 (11.9)	67.9 (11.3)	0.03	66.1 (11.9)	0.01
Femoral neck BMD (g/cm^2^)	0.83 (0.12)	0.79 (0.11)	<0.001	0.75 (0.11)	<0.001
Femoral neck T-scores	–1.46 (1.19)	–1.89 (1.10)	<0.001	–2.30 (1.06)	<0.001
Prior fracture, n (%)			0.004		0.11
0	972 (75.9)	242 (68.0)		60 (68.2)	
1	185 (14.4)	68 (19.1)		16 (18.2)	
2	89 (7.0)	26 (7.3)		6 (6.8)	
3	35 (2.7)	20 (5.6)		6 (6.8)	
Fall in the last 12 month, n (%)			0.03		0.02
0	903 (70.5)	228 (64.0)		52 (59.1)	
1	360 (28.1)	125 (35.1)		36 (40.9)	
2	18 (1.4)	3 (0.8)		0 (0)	
**Men**	**(n = 1238)**	**(n = 117)**		**(n = 47)**	
Age (y)	69.6 (5.6)	71.1 (6.5)	0.006	72.8 (5.9)	<0.001
Height (cm)	174.2 (6.6)	176.1 (6.4)	0.002	176.7 (6.8)	0.008
Weight (kg)	80.5 (11.5)	83.0 (13.8)	0.02	82.4 (14.6)	0.26
Femoral neck BMD (g/cm^2^)	0.94 (0.13)	0.88 (0.13)	<0.001	0.84 (0.1)	<0.001
Femoral neck T-scores	–0.91 (1.22)	–1.40 (1.18)	<0.001	–1.74 (0.88)	<0.001
Prior fracture, n (%)			0.057		0.12
0	1119 (90.4)	101 (86.3)		38 (80.9)	
1	90 (7.3)	10 (8.6)		7 (14.9)	
2	21 (1.7)	6 (5.1)		2 (4.2)	
3	8 (0.7)	0 (0)		0 (0)	
Fall in the last 12 month, n (%)			0.16		0.63
0	856 (69.1)	71 (60.7)		32 (68.1)	
1	361 (29.2)	44 (37.6)		15 (31.9)	
2	21 (1.7)	2 (1.7)		0 (0)	

Values are mean (SD), unless otherwise specified.

* Compared with non-fracture group.

**Figure 1:**
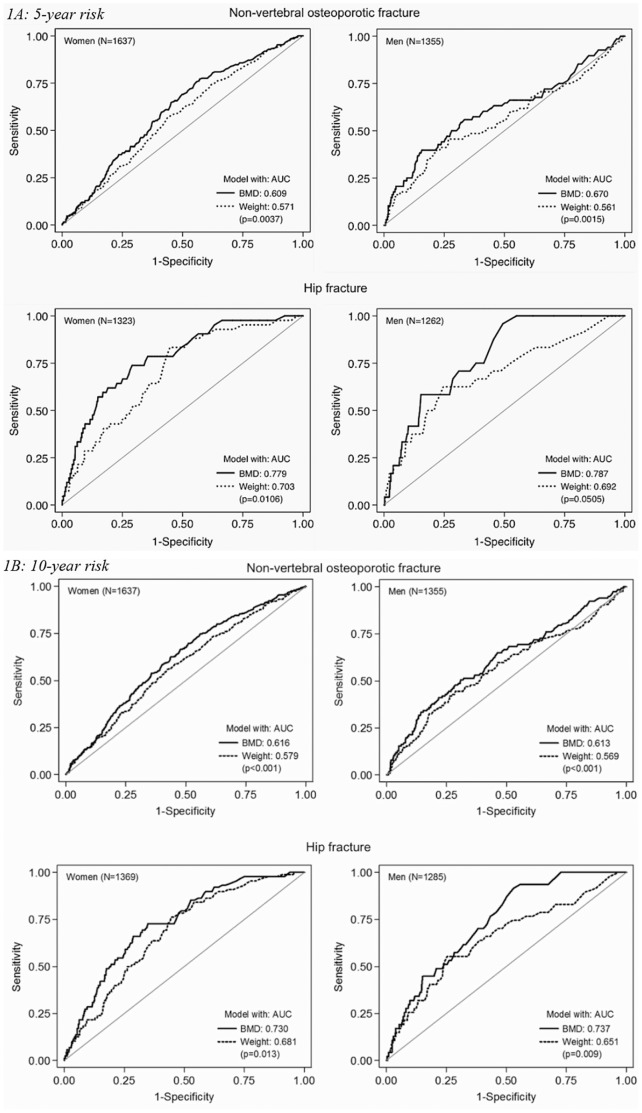
Receiver Operating Characteristic (ROC) curves for model with BMD (continuous line) and model with weight (dashed line) for non-vertebral osteoporotic fracture (upper panel) and hip fracture (lower panel) in women and men based on (1A): 5-year predicted risk, and (1B): 10-year predicted risk.

The area under receiver operating characteristics curve (AUC) illustrates the prognostic discrimination for non-vertebral osteoporotic and hip fractures of both models (Figure 1A and 1B). The AUCs for both models were higher for hip (ranging from 0.73 to 0.79) than non-vertebral osteoporotic fractures (AUC 0.61–0.67) with the highest AUC in the 5-year risk analyses. Moreover, the AUCs for the model with BMD were significantly higher than the model with weight for both fracture types among both women and men (all *p*<0.05).

**Figure 2:**
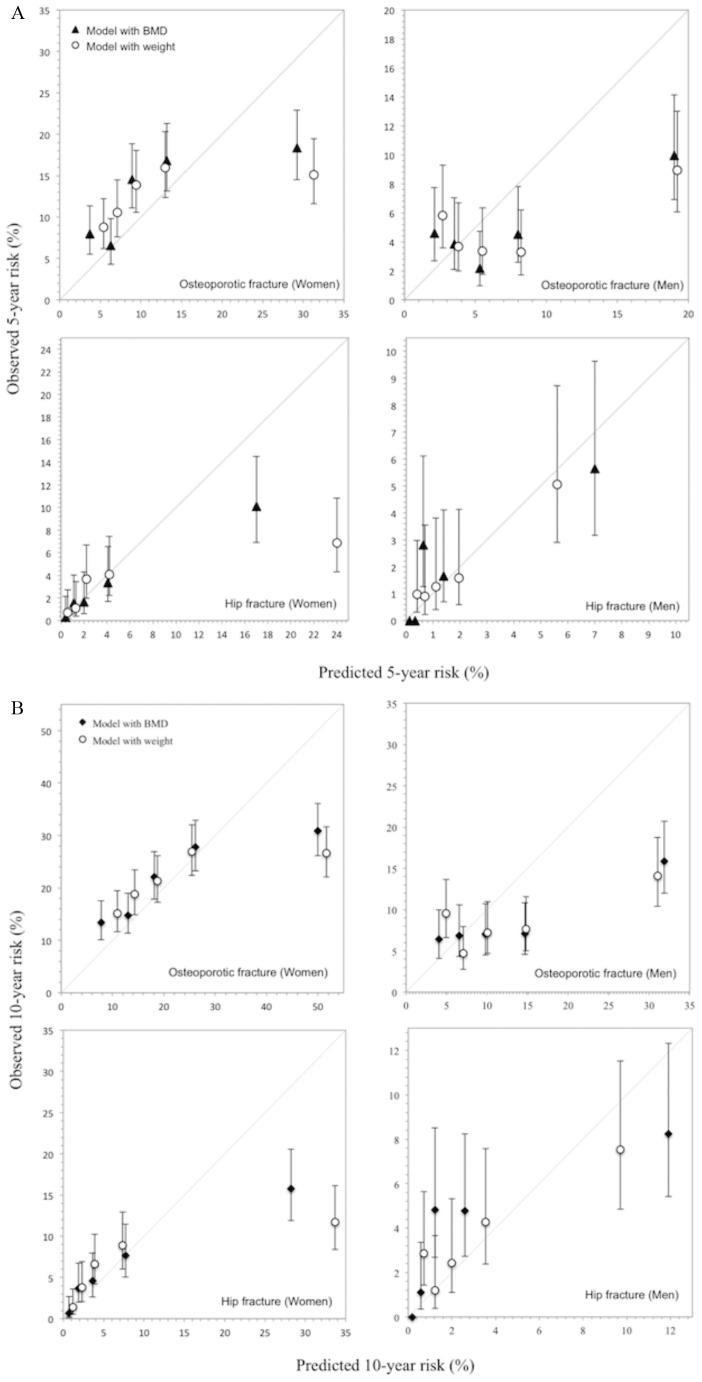
Concordance between the predicted and observed risk of non-vertebral osteoporotic fracture (upper panel) and hip fracture (lower panel) in the Tromsø Study cohort, according to the Garvan nomograms. (A): Quintile cut-offs for the predicted 10-year risk (%) of non-vertebral osteoporotic fracture in women were: 10.8, 15.3, 21.2 and 31.9 for model with BMD (M1); and 12.5, 16.3, 21.3 and 31.5 for model with weight (M2). Corresponding cut-offs in men were 5.3, 8.0, 11.7 and 18.3 for M1; and 5.9, 8.3, 12.1, 17.9 for M2. Quartile cut-offs for the predicted 10-year risk (%) of hip fracture in women were: 1.3, 2.6, 4.9 and 11.2 for M1 and 1.7, 2.9, 5.0 and 11.1 for M2; In men, 0.3, 0.8, 1.6 and 3.9 for M1; and 0.9, 1.5, 2.6 and 4.8 for M2. (B): Quintile cut-offs for the predicted 5-year risk (%) of non-vertebral osteoporotic fracture in women were: 5.2, 7.4, 10.5 and 16.4 for model with BMD (M1); and 6.2, 8.1, 10.8 and 16.5 for model with weight (M2). Corresponding cut-offs in men were 2.8, 4.2, 6.3 and 10.0 for M1; and 3.2, 4.5, 6.6, 10.1 for M2. Quartile cut-offs for the predicted 5-year risk (%) of hip fracture in women were: 0.7, 1.4, 2.7 and 5.8 for M1 and 0.9, 1.6, 2.8 and 6.3 for M2; In men, 0.2, 0.4, 0.8 and 2.1 for M1; and 0.5, 0.8, 1.4 and 2.7 for M2.

With respect to predictive accuracy of the two models (Figure 2A and 2B), there was a close agreement between predicted and observed risk of fracture, with higher concordance between predicted and observed risk in general for women than for men. In women and men with fracture risk in the highest quintile, both BMD and weight models over-estimated the 5-year and 10-year risks of fracture. Moreover, both the 5-year and the 10-year probability of fracture in those with fracture were on average consistently higher than in those without fracture for both models. The 10-year probability analyses showed that in women, the ratios of predicted risk of non-vertebral osteoporotic fracture between fracture and non-fracture groups were 1.30 (95% CI 1.20–1.40) and 1.16 (1.09–1.24) for BMD and weight models, respectively. The corresponding ratios for hip fracture were, respectively, 2.80 (2.12–3.70) and 2.02 (1.58–2.59). Similar results were obtained in men; for non-vertebral osteoporotic fracture 1.36 (1.19–1. 56) and 1.19 (1.05–1.34) for BMD and weight models, respectively and for hip fracture 3.10 (2.08–4.62) and 1.67 (1.17–2.28).

Models with BMD performed better than models with weight in terms of correct reclassification of fracture and non-fracture subjects in their risk groups in women and in men (Table 2). Compared to the model with BMD, the model with weight showed a net decrease of 9.6% in women and 17.1% in men, in reclassifying non-vertebral osteoporotic fracture cases as “high risk” group, and a decrease of 1.1% in women and 0.1% in men in reclassifying non-fracture subjects as “low risk” group. The overall net decrease in reclassification of the model with weight was 10.6% (*p* = 0.008) in women and 17.2% (*p* = 0.001) in men. For hip fracture, there was no significant difference between the two models. The overall reclassification index showed a net decrease of 13.3% (*p* = 0.07) in women and 17.5% (*p* = 0.09) in men for the model with weight compared to the model with BMD.

**Table 2:** Net Reclassification Improvement (NRI) of model with body weight compared to model with BMD.

	Women			Men		
	Index	(SE)	*p*-value	Index	(SE)	*p*-value
**Osteoporotic fracture**						
NRI for fracture	–0.096	0.036	*0.008*	–0.171	0.049	*0.0009*
NRI for non-fracture	–0.011	0.018	*0.536*	–0.001	0.017	*0.961*
NRI overall	–0.106	0.040	*0.008*	–0.172	0.052	*0.001*
**Hip fracture**						
NRI for fracture	–0.125	0.070	*0.078*	–0.191	0.098	*0.061*
NRI for non-fracture	–0.008	0.019	*0.676*	0.016	0.021	*0.450*
NRI overall	–0.133	0.072	*0.070*	–0.175	0.100	*0.093*

*Values are differences in proportion of correct classification between the models with weight and BMD in each category. Negative values showed that the model with BMD performed better than the model with weight and vice versa.*

## Discussion

This study validated the Garvan nomograms in a new population with a substantially higher fracture risk. The nomograms were valid and reasonably accurate in identifying individuals at high risk of fracture in this population. The models with BMD performed better than those with body weight in fracture prediction.

The assessment of fracture risk is moving toward the absolute risk approach, in which an individual’s risk is estimated based on the individual’s unique risk profile. The individualization of risk can help make decision concerning treatment for a patient. A number of fracture risk assessment tools have been developed, and among the most popular algorithms are the World Health Organization’s FRAX and Garvan Fracture Risk Calculator. These algorithms have been widely validated in independent populations. A recent review of 13 tools for prediction of fractures found that the Garvan model performed as good as or better than more complex models [5]. Compared to other tools, the Garvan nomogram is easy to use without complex computation or the need of computer software which can be impractical or inaccessible in primary care settings [9]. Although the nomograms incorporate fewer number of risk factors compared to other prediction tools, their good predictive performance might be attributed to the strong contribution of the cumulative effect of history of previous fracture and falls on fracture risk [12].

Our findings of moderate discriminative performance of the nomograms with BMD are similar to those reported earlier on the 10-year prediction model. In New Zealand postmenopausal women followed more than 8 years, the Garvan nomograms had AUC values of 0.64 for osteoporotic fractures and 0.67 for hip fractures [14]. In the Global Longitudinal Study of Osteoporosis in Women (GLOW) study (including 60+ years old women from 10 countries with 2 years follow-up), the AUC was 0.64 for osteoporotic fractures and 0.61 for hip fractures [15]. In a Canadian cohort of women and men followed more than 8 years, the discrimination was assessed using the Harrell’s C statistics (analogous to AUC) and found to be 0.69 in women and 0.70 in men for low-trauma fractures, and 0.80 in women and 0.85 in men for hip fractures [13]. In addition to previous validations, the current validation also tested the performance of a model with body weight instead of BMD. Overall, the discrimination values for the model with weight were lower than the model with BMD for both fracture types in women and in men. Nonetheless, the model with weight showed a modest performance for hip fractures.

The discriminative value (AUC) of a model does not reflect its clinical value, however evaluation of calibration of prediction models is important for the translation to clinical practice [22]. Similar to previous validations [13], [14], this study showed very good calibration of the nomograms, particularly in women in the four lower quintiles of risk. Although the nomograms (with BMD or body weight) over-estimated the risk of fracture in high risk individuals, these individuals would be candidates for intervention in any case. In fact their outcomes may have been modified by treatment received. However, data on treatment were not available in the present study. Compared to women and men in the lower risk quintiles, those in the highest risk quintile were older and had shorter mean follow-up, indicating an increased competing risk of death and thus potentially lower observed risk. In addition, the predicted 10-year risk was compared with an observed risk of shorter duration (mean follow-up 6.9–7.1 years), although similar effects were observed in the 5-year risk analyses. However, possibility of starting osteoporosis treatment during follow-up or model shrinkage – models’ tendency to overestimate when using independent data– could contribute to the over-estimates [10], [13]. Nonetheless, the nomograms overall predictive ability at the individual level can potentially be useful in clinical practice and as a measure of severity of osteoporosis for the identification of patients in need to be on anti-osteoporosis treatment, and even can be used for selecting patients for clinical trials [9].

This study provides the first external evaluation of performance of the model with body weight compared to model with BMD. The model with BMD performed better in reclassifying both those with and without fracture. The decrease in reclassification for the model with weight is attributed to the overall better sensitivity and specificity of the model with BMD. The reclassification analysis is useful for comparison of the two models in the same group of patients, but not for necessarily for assessment of the models’ clinical utility [23]. However, the high predictive accuracy of the model with weight demonstrated by the calibration performance indicates its validity in clinical settings where BMD measurements may not be readily available.

Strengths of this validation analysis include the prospective population-based design with a long follow-up of a large cohort of women and men, with a validated fracture registry capturing all non-vertebral fractures in the cohort. This gave the opportunity to examine the nomograms performance in a similar study design as the one in which the nomograms were developed but in a distinct independent cohort in a distinct geographic location. Limitations of the study included the lack of vertebral fracture registration, the identification of the energy involved (i.e. low versus higher trauma) in all of the fractures, and data on treatment during follow-up, which would have strengthened the validation. Furthermore, the results cannot be extrapolated to younger women and men, and because of lack of certain data, it was not possible to make performance comparisons between the nomograms and the widely used FRAX tool [4], [5].

In conclusion, the Garvan nomograms were valid and clinically accurate in discriminating between fracture and non-fracture subjects in an independent Norwegian cohort of women and men supporting the robustness of the algorithms. Models with BMD performed better than those with body weight in fracture prognosis. Although the nomograms somewhat over-estimated the risk of fracture in high risk individuals, their predictive ability would be useful in clinical practice.

## Supporting Information

Appendix S1 **The Garvan Fracture Risk Calculator equations for estimating 5-year and 10-year risks of hip and any fracture in women and men.**
(PDF)
