# Supplementary material for: External Validation of the Garvan Nomograms for Predicting Absolute Fracture Risk: The Tromsø Study
Source: PLoS One. 2014 Sep 25;9(9):e107695. doi: 10.1371/journal.pone.0107695 (PMC4177811; doi:10.1371/journal.pone.0107695)
Supplement: Appendix S1 — The Garvan Fracture Risk Calculator equations for estimating 5-year and 10-year risks of hip and any fracture in women and men. (PDF) [file pone.0107695.s001.pdf]

Methods for estimating 5-year and 10-year risks of hip and any fracture

The prognostic models for hip [1] and any fracture [2] were developed for men and women separately. The models were developed by making use of data from the Dubbo Osteoporosis Epidemiology Study. The Study, which began in 1989, has followed more than 2000 men and women for more than 20 years. All individuals aged at least 60 years old at the study entry. After extensive analyses and consideration of more than 50 risk factors, we found that 5 factors markedly affected fracture outcome: age, bone mineral density, body weight, a history of prior fracture after the age of 50, and any falls during the past 12 months. These risk factors were then used to develop and internally validate the prognostic model.

The cumulative risk of fracture at time *t* of an individual, denoted by *risk*(*t*), conditioned on the individual's risk profile is estimated by the following equation:

$$risk(t) = 1 - S_0(t)^{\exp(\gamma)}$$

Where $S_0(t)$ is the baseline risk, with actual values being shown in **Table 1**:

Table 1: $S_0(t)$ for 5-year and 10-year period by fracture type and gender

	Any fracture		Hip fracture	
	**Women**	**Men**	**Women**	**Men**
5 years	0.995750118	0.999921076	0.999896685	0.999998862
10 years	0.990905138	0.999848975	0.999793070	0.999997778

γ is a linear function that takes into account the risk profile for the individual

$$\gamma = a \times \text{Age} + b \times \text{Tscore} + c \times \text{Priorfx} + d \times \text{Falls} \quad (1)$$

or

$$\gamma = a \times \text{Age} + b \times \text{Weight} + c \times \text{Priorfx} + d \times \text{Falls} \quad (2)$$

where (1) is the model with BMD and (2) is the model with body weight replaced for BMD; *a, b, c, d* are coefficients associated with a risk factor; **Age** is expressed in years; **Tscore** is the actual femoral neck BMD T-score; **Priorfx** is the number fractures from the age of 50

years (0 for no prior fracture, 1=1, 2=2 , and 3 for >= 3 fractures); and **Falls** is the number of falls during the past 12 months (0 for no fall,1=1, 2=2 , and 3 for >= 3 falls in the last 12 months). The actual values *a, b, c, d* are shown in **Table 2** (in case of BMD) or **Table 3** (in case of weight).

Table 2: Coefficients associated with each factor by fracture type and gender for the models with femoral neck BMD

	Any fracture		Hip fracture	
	**Women**	**Men**	**Women**	**Men**
Age (*a*)	0.0321	0.0883	0.0507	0.107
Tscore (*b*)	-0.4022	-0.2986	-0.8417	-1.007
Prior fx (*c*)	0.5691	0.8454	0.8127	0.599
Fall (*d*)	0.2038	0.0981	0.3614	0.211

Table 3: Coefficients associated with each factor by fracture type and gender for the models with body weight

	Any fracture		Hip fracture	
	**Women**	**Men**	**Women**	**Men**
Age (*a*)	0.0511	0.09071	0.0866	0.138
Weight (*b*)	-0.0099	-0.00765	-0.0444	-0.008
Prior fx (*c*)	0.6709	1.00818	1.1916	0.854
Fall (*d*)	0.1985	0.12797	0.2946	0.233

Example: A 60 year-old woman with femoral neck BMD T-score = -2.5, had a history of one prior fracture and one fall during the past 12 months. Her 10-year risks of hip fracture can be estimated as follows:

Step 1: Determine the baseline probability of no fracture in Table 1

For 10-year risk: $S_0(10) = 0.999793070$

Step 2: Compute the linear term by using coefficients in Table 2

$$\begin{aligned}\gamma &= a \times \text{Age} + b \times \text{Tscore} + c \times \text{Priorfx} + d \times \text{Falls} \\ &= (0.0507\times60) + (-0.8417\times-2.5) + (0.8127\times1) + (0.3614\times1) \\ &= 6.32035\end{aligned}$$

Step 3: Compute the t-year risk of hip fracture

$$risk(10) = 1 - 0.999793070^{\exp(6.32035)} = 0.109$$

In other word, the woman's 10-year of hip fracture is 10.9%.

References

[1] Nguyen, N. D., Frost, S. A., Center, J. R., Eisman, J. A., and Nguyen, T. V. Development of a nomogram for individualizing hip fracture risk in men and women. Osteoporos Int 2007; 18:1109-17.

[2] Nguyen, N. D., Frost, S. A., Center, J. R., Eisman, J. A., and Nguyen, T. V. Development of prognostic nomograms for individualizing 5-year and 10-year fracture risks. Osteoporos Int 2008; 19:1431-44.
